# Familial Carney complex with embolic ischemic stroke: a case report and literature review

**DOI:** 10.3389/fonc.2025.1590877

**Published:** 2025-08-18

**Authors:** Tingting Luo, Mengxing Yin, Lijin Li, Mengjiao Sun, Zhen Li, Pengge Li, Mengmeng Liu, Xinxin Sun, Zhanyi Wu, Yifan Liu, Lulu Zhang, Zhiyao Wang, Suyun Hou, Shaohua Hua

**Affiliations:** Department of Ultrasound, The First Affiliated Hospital of Zhengzhou University, Zhengzhou, China

**Keywords:** Carney complex, myxoma, embolic ischemic stroke, PRKAR1A, multiple endocrine neoplasia

## Abstract

**Background:**

Carney complex (CNC) is a rare autosomal dominant multiple neoplasia syndrome characterized by cutaneous and mucosal pigmented lesions, cardiac myxomas, and various endocrine and non-endocrine tumors.

**Methods:**

We report a familial case of CNC presenting initially with embolic ischemic stroke. Comprehensive clinical evaluation, imaging studies, histopathological examination, and genetic analysis were performed on the proband and family members, with a literature review summarizing the clinical features of CNC.

**Results:**

A pathogenic *PRKAR1A* mutation was identified in affected family members, who exhibited typical clinical features of CNC. The proband presented with stroke secondary to cardiac myxoma, highlighting the importance of early recognition in patients with multi-system manifestations.

**Conclusions:**

CNC should be suspected in young patients presenting with cardiac myxoma and stroke. Clinicians should maintain high vigilance for patients with multi-system symptoms and complex family histories. Comprehensive family history assessment and genetic testing facilitate early diagnosis of CNC.

## Introduction

Carney complex (CNC) is a rare multiple system tumor syndrome. During the 1970s-1980s, this disease existed in forms such as LAMB syndrome and NAME syndrome, which were later confirmed to represent different phenotypes of the same disease (1, 2). Approximately 750 confirmed cases have been reported globally; however, the true prevalence remains to be elucidated due to underdiagnosis and phenotypic heterogeneity (3).

The pathogenesis of CNC centers on dysregulation of the cAMP-PKA signaling pathway. *PRKAR1A* encodes the type I regulatory subunit α of protein kinase A, and its loss-of-function mutations result in excessive activation of catalytic subunits, driving hyperproliferation of cAMP-sensitive tissue cells and causing neoplastic lesions in related organs (4). To date, more than 130 pathogenic *PRKAR1A* variants have been identified in over 400 families (5). Additionally, genes such as *PRKACA*, *PDE11A*, and *PDE8B* are involved in pathogenesis, suggesting that CNC involves synergistic disruption of multiple signaling pathways (6, 7).

However, the rarity and phenotypic heterogeneity of CNC continues to pose diagnostic challenges. When patients present with few typical symptoms or have unclear family histories, key diagnostic clues are easily overlooked, leading to delayed diagnosis. This study reports a familial case of CNC presenting initially with ischemic stroke, aiming to emphasize the importance of detailed family history collection and multi-system evaluation in early diagnosis.

## Case presentation

A 14-year-old female patient presented to the local hospital with acute onset of aphasia and right-sided hemiplegia following a fall. She was diagnosed with severe closed traumatic brain injury complicated by multiple cerebral infarctions. Echocardiography performed during the initial hospitalization revealed two myxomas in the left atrium, suggesting cardiogenic cerebral embolism. Unfortunately, the cardiac lesion was initially treated merely as an isolated finding, without consideration of an underlying genetic syndrome. Four months later, the patient was transferred to our hospital for cardiac surgery, and postoperative pathology confirmed the diagnosis of myxoma. Considering that atrial myxoma in young patients is an important indicator of Carney complex, we immediately conducted a systematic evaluation, including detailed cutaneous and mucosal examination and family history investigation. Genetic testing revealed a pathogenic *PRKAR1A* mutation, ultimately confirming the diagnosis of CNC.

### Physical examination

**Figure 1:**
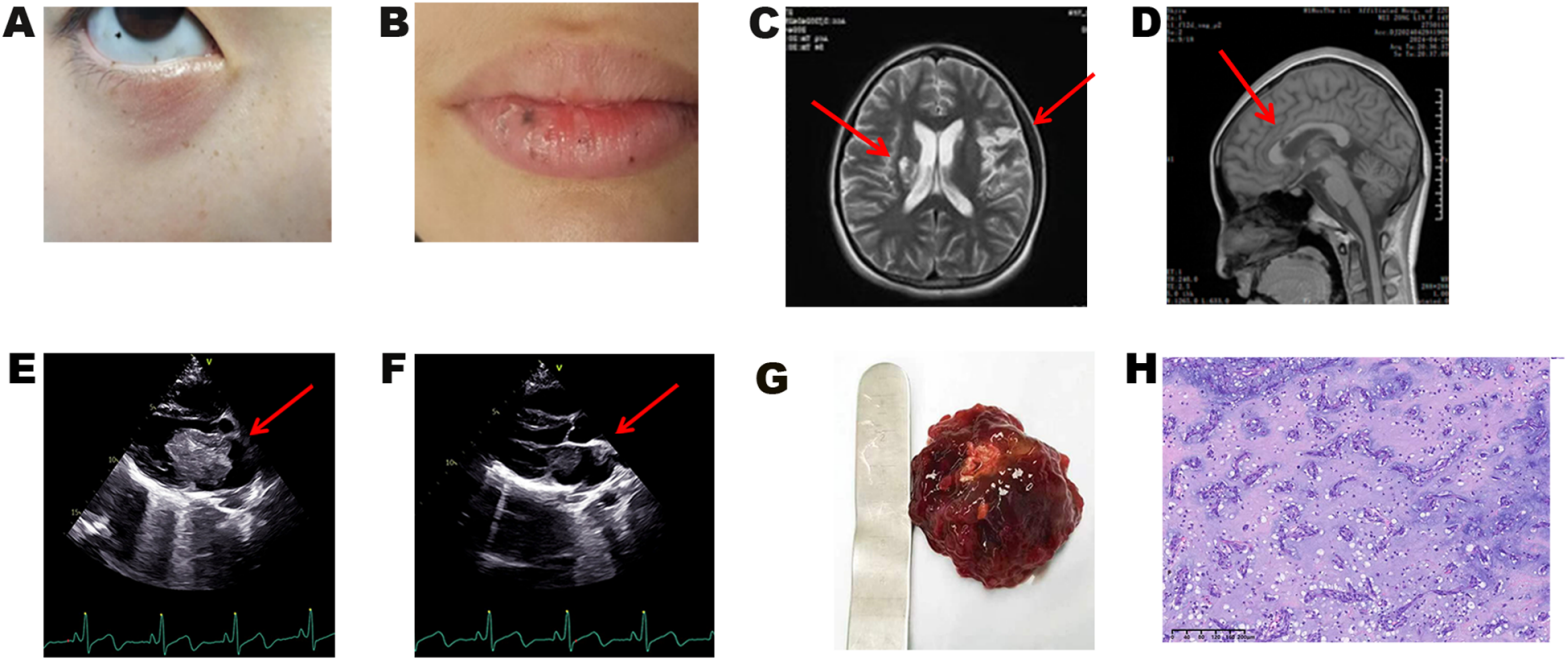
Clinical manifestations and pathological results of the proband. **(A)** Characteristic lentigines on the eyelids, conjunctiva, and facial regions, and **(B)** on the vermilion border of the lips and oral mucosa. **(C, D)** Brain MRI demonstrating multiple cerebral infarctions. **(E, F)** Transthoracic echocardiography showing **(E)** left atrial myxoma and **(F)** myxoma at the left atrial appendage orifice. **(G)** Gross specimen of the resected cardiac myxoma. **(H)** Histopathological examination of cardiac myxoma (H&E staining).

Upon admission, vital signs were stable (temperature 36.5°C, pulse 76 beats per minute, respiratory rate 20/min, blood pressure 125/74 mmHg, BMI 17.6 kg/m²). Dermatological examination revealed characteristic punctate pigmentation involving the lips, conjunctiva, periorbital regions, and vulvar mucosa (**Figures 1A, B**). Cardiovascular examination demonstrated regular heart rhythm with a grade 2/6 systolic ejection murmur audible at the left sternal border. Neurological examination showed right-sided hemiplegia with upper limb muscle strength 4/5 and lower limb muscle strength 2/5 according to the Medical Research Council scale. Other system examinations were unremarkable.

### Laboratory findings

Laboratory investigations revealed elevated N-terminal pro-B-type natriuretic peptide (NT-proBNP) at 469.00 pg/mL (normal <125 pg/mL) and elevated high-sensitivity cardiac troponin T (hs-cTnT) at 0.110 ng/mL (normal <0.014 ng/mL). D-dimer was 0.44 mg/L (normal <0.50 mg/L). Complete blood count, comprehensive metabolic panel (including blood glucose, electrolytes, liver and kidney function), and thyroid function tests were all within normal limits.

### Imaging studies

Brain magnetic resonance imaging (MRI) demonstrated multiple cerebral infarctions (**Figures 1C, D**). Transthoracic echocardiography revealed two masses within the left atrium: the primary mass measured 56 × 33 × 39 mm, presenting as a moderately echogenic lesion with well-defined but irregular borders, relatively homogeneous internal echoes, and no obvious calcification or necrotic changes. The mass was attached to the atrial septum at the fossa ovalis via a pedicle, exhibited significant oscillatory motion with the cardiac cycle, prolapsed through the mitral valve into the left ventricle during diastole, and returned to the left atrium during systole, resulting in functional mitral stenosis. The secondary mass was located at the left atrial appendage orifice, measuring 11 × 10 mm, with moderate echogenicity, clear borders, and homogeneous internal echoes (**Figures 1E, F**). Differential diagnosis included atrial thrombus (typically hypoechoic, non-pedunculated, with poor mobility), primary cardiac lymphoma (commonly involving the right atrium with poorly defined borders and heterogeneous echogenicity), and other cardiac tumors. Based on the characteristic attachment to the atrial septum at the fossa ovalis, pedunculated connection, and typical movement pattern, the echocardiographic findings supported the diagnosis of atrial myxoma. Ultrasonography also revealed bilateral thyroid cystic nodules, while other organs (ovaries, adrenal glands, breast, pancreas, hepatobiliary system) showed no abnormalities.

### Surgical treatment and pathological findings

The patient underwent thoracoscopic cardiac mass resection to prevent recurrent cerebral and systemic embolism. The surgery was performed under general anesthesia through a minimally invasive right fourth intercostal space approach. Cardiopulmonary bypass was established via arterial cannulation through the right femoral artery and venous cannulation through the right femoral vein. Cardioplegic solution was infused through the ascending aortic root to induce cardiac arrest, ensuring myocardial protection. Following left atriotomy, the myxoma was completely excised with a 3 mm margin around the tumor pedicle to prevent tumor fragmentation and embolization. Electrocoagulation was used to thoroughly cauterize the tumor attachment site to ensure complete tissue removal. Transesophageal echocardiography confirmed complete excision with no residual tissue or complications.

Two left atrial myxomas were successfully and completely resected. The larger mass (50 × 50 × 50 mm) originated from the atrial septum at the fossa ovalis, while the smaller mass (10 × 10 × 10 mm) was attached to the base of the left atrial appendage. Gross examination revealed typical characteristics: gray-red, multilobulated masses with a gelatinous texture and focal hemorrhage (**Figure 1G**). Histopathological examination demonstrated typical myxoma features with stellate and spindle cells distributed within a myxoid matrix, confirming the diagnosis of cardiac myxoma (**Figure 1H**). Postoperative echocardiography showed complete tumor resection with no residual lesions. The patient recovered uneventfully without postoperative complications. Regular cardiac monitoring and systematic screening for CNC-related manifestations were performed according to established protocols (3) (**Supplementary Table 1**).

### Whole exome sequencing

In this study, peripheral venous blood samples (2 mL each) were collected from the proband and both parents for genetic analysis. Whole exome sequencing (WES) was employed to detect coding sequence variants in the proband, revealing a pathogenic variant c.491_492delTG (p.Val164fs) in the PRKAR1A gene. Subsequently, PCR-Sanger sequencing was performed to validate this variant in both parents. The results demonstrated that this pathogenic variant was detected in the father, while the mother tested negative (**Table 1**).

**Table 1:** Genetic analysis results in the proband and family members.

Gene	Transcript	Variant	Protein Change	Zygosity	MAF	Classification	Inheritance Pattern	REF/ALT	REVEL	Source
*PRKAR1A*	NM_001369390	c.491_492delTG	p.Val164fs	het	3.98e-6(0)	LP	AD	80/62	–	Paternal

fs, frameshift; het, heterozygous; LP, likely pathogenic; AD, autosomal dominant; MAF, minor allele frequency; REF/ALT, reference/alternative allele; REVEL, Rare Exome Variant Ensemble Learner; -, not applicable or not detected.

### Family history and clinical manifestations

**Figure 2:**
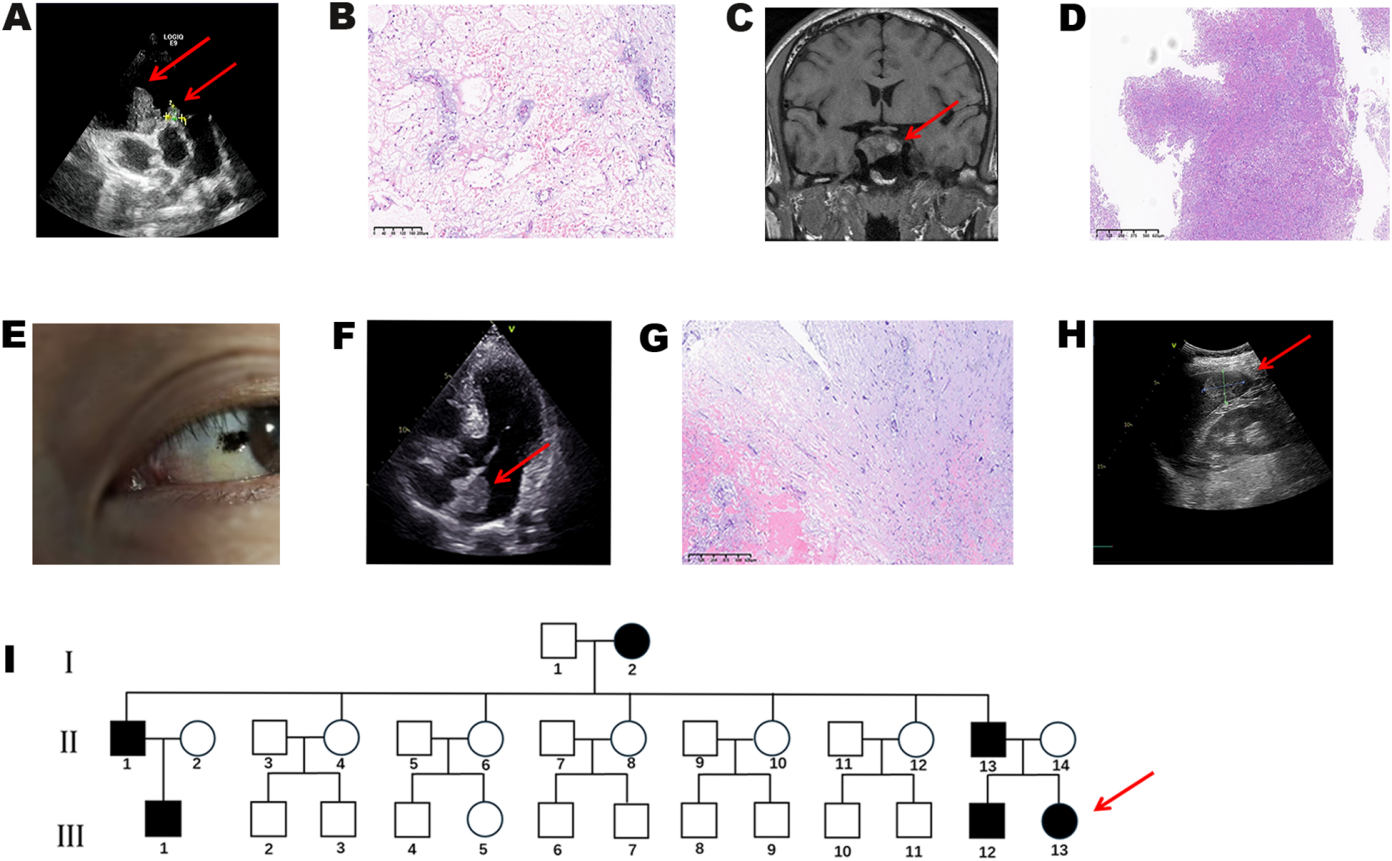
Clinical and genetic characteristics of affected family members. **(A)** Echocardiography of the patient’s older brother showing two right ventricular myxomas. **(B)** Histopathological examination of the brother’s cardiac myxoma (H&E staining). **(C)** Brain MRI of the brother demonstrating a sellar mass (15 × 28 × 17 mm). **(D)** Histopathological examination of the brother’s pituitary adenoma (H&E staining). **(E)** Conjunctival lentigines in the father. **(F)** Echocardiography of the patient’s father showing left atrial myxoma (30 × 20 mm). **(G)** Histopathological examination of the father’s cardiac myxoma (H&E staining). **(H)** Ultrasound image of hepatic hemangioma in the patient’s father. **(I)** Three-generation pedigree. Solid symbols: confirmed CNC patients; hollow symbols: unaffected individuals; circles: females; squares: males; arrow: proband.

Further family history investigation revealed that the patient’s grandmother (I2) and older brother (III12) both had a history of cardiac myxoma (**Figures 2A, B**), while the father (II13) had a history of adrenocortical adenoma. The patient’s paternal uncle (II1) died from thyroid malignancy, and her paternal cousin (III1) underwent unilateral orchiectomy for testicular malignancy. Based on the family history, systematic screening was conducted among family members. Screening revealed that the patient’s older brother (III12) was tall in stature (192 cm) with significantly elevated growth hormone (GH) levels (90.11 ng/mL) and gynecomastia. Brain MRI with and without contrast demonstrated a sellar mass (**Figure 2C**), and postoperative pathology confirmed pituitary adenoma (**Figure 2D**). The patient’s father exhibited characteristic punctate pigmentation (**Figure 2E**) and was found to have a left atrial myxoma (**Figure 2F**). Thoracoscopic resection was performed, and postoperative pathology confirmed cardiac myxoma (**Figure 2G**). Additionally, hepatic hemangioma was detected in the patient’s father (**Figure 2H**). According to the CNC diagnostic and supplemental criteria established by Stratakis et al (8) (**Supplementary Table 2**), a family pedigree was constructed (**Figure 2I**).

### Literature review

#### Cutaneous manifestations

Cutaneous lesions represent the earliest and most characteristic manifestations of CNC, occurring in over 80% of patients. Lentiginous nevi are primarily distributed on the lips, conjunctiva, genitalia, and other sites, appearing and worsening before puberty (9). Epithelioid blue nevi are rare in the general population but demonstrate significantly increased incidence in CNC patients (10). Cutaneous myxomas occur in 30-55% of cases, predominantly affecting the eyelids, external auditory canal, and nipples (9).

#### Cardiac involvement

Cardiac myxomas represent the most severe clinical manifestation and leading cause of death in CNC, with an incidence of 42.6%-63.1%, and more than half of patients experience recurrence (11, 12). Unlike sporadic myxomas, CNC-associated myxomas can involve any cardiac chamber, occur at a younger age, and demonstrate equal incidence between males and females (11).

#### Endocrine abnormalities

Among endocrine manifestations, primary pigmented nodular adrenal disease (PPNAD) is most common, affecting 26-60% of patients. Its pathological features include multiple pigmented micronodules within the adrenal cortex with autonomous cortisol secretion, leading to ACTH-independent Cushing syndrome (5, 13). Pituitary dysfunction occurs in 30-75% of patients, with clinical presentations ranging from subclinical growth hormone elevation to overt acromegaly, with 15%-18.9% of patients developing acromegaly (14). Thyroid involvement affects up to 60% of patients, with nodular thyroid disease being particularly common in children and adolescents. Although most lesions are benign follicular adenomas or cystic lesions, the incidence of thyroid malignancy (papillary or follicular carcinoma) is approximately 10% (15).

#### Other manifestations

Other important manifestations include psammomatous melanotic schwannomas (PMS), observed in approximately 10% of patients, which predominantly affect gastrointestinal nerves, paravertebral sympathetic chains, and chest wall, with risk of malignant transformation (3, 16). Large cell calcifying Sertoli cell tumors (LCCSCT) affect 20%-50% of male patients and can cause gynecomastia, precocious puberty, and reproductive dysfunction (3, 17). Female patients commonly develop breast myxomas and ductal adenomas, with significantly elevated risk of breast cancer (18).

## Discussion

Based on our case series and literature review, several key clinical features should alert clinicians to the possibility of Carney complex in patients presenting with cardiac myxomas. Young patients (particularly adolescents and young adults) presenting with cardiac myxomas in atypical anatomical locations, multiple or recurrent myxomas, cutaneous pigmentary abnormalities, concurrent endocrine dysfunction, or positive family history should be considered high-risk for Carney complex and require comprehensive diagnostic evaluation (8).

Cardiac myxomas represent the major life-threatening complication in CNC patients, potentially causing embolic events (stroke, peripheral arterial embolism), blood flow obstruction, and sudden cardiac death (19). Therefore, early screening and timely surgical intervention are crucial. Surgical resection is the standard treatment, requiring wide-margin excision techniques, active prevention of intraoperative embolization, and preparation for multiple surgeries due to potential recurrent lesions (12). Our patient presented with embolic stroke as the initial manifestation, with multiple cerebral infarctions leading to aphasia and right-sided hemiplegia, ultimately leading to the diagnosis of CNC-associated cardiac myxoma, illustrating the insidious and severe nature of this disease.

Based on the practical experience gained from this study and existing literature evidence, we recommend prioritizing the implementation of comprehensive multigene panels that incorporate all known CNC-associated genes with copy number variation detection capabilities as the first-line screening strategy in clinical practice. This approach provides more comprehensive coverage of the genetic heterogeneity inherent in the disease (5, 6). For suspected cases with negative multigene panel results but clinical presentations highly suggestive of CNC, further consideration should be given to employing whole exome sequencing (WES) or whole genome sequencing (WGS) for more in-depth genomic analysis (20), to identify potential novel pathogenic variants or rare genetic mechanisms. The detection of hepatic hemangioma in our patient’s father suggests that hepatic involvement may represent an important but underestimated clinical manifestation of CNC. Previous studies have demonstrated that hepatic involvement occurs in up to 50% of CNC patients carrying pathogenic PRKAR1A variants (21), indicating a significant association between the two. Therefore, we recommend incorporating hepatic imaging screening into routine surveillance protocols for CNC patients, particularly those carrying PRKAR1A mutations (3).

In conclusion, this study successfully reported a familial CNC case and confirmed the pathogenic role of PRKAR1A mutations. However, we must objectively acknowledge the limitations of this study across multiple dimensions. At the genetic testing level, our analysis was primarily focused on the PRKAR1A gene and did not encompass comprehensive screening of all CNC-associated candidate genes, while lacking the analytical capacity for complex genetic variations such as copy number variations and structural rearrangements. In terms of pathological investigation, our histological analysis was confined to routine pathological diagnosis, lacking exploration of deeper biological characteristics such as pluripotent cardiac stem cell markers (22), which may have constrained our comprehensive understanding of the origin and pathogenesis of cardiac myxoma. At the translational clinical level, constrained by the sample size limitations and insufficient analytical depth inherent in single-case studies, we were unable to achieve breakthrough progress in diagnostic biomarker development. Despite these aforementioned limitations, our findings still underscore the significant value of familial screening and genetic counseling in early diagnosis, risk assessment, and disease management of CNC, providing valuable references for standardized diagnosis and treatment of CNC in clinical practice.

## Data Availability

The data contain sensitive patient information, including clinical and genetic details. Therefore, the raw data cannot be made publicly available to protect patient confidentiality. Requests to access these datasets should be directed to hsh1852@126.com.
