# Supplementary material for: Familial Carney complex with embolic ischemic stroke: a case report and literature review
Source: Front Oncol. 2025 Aug 18;15:1590877. doi: 10.3389/fonc.2025.1590877 (PMC12399879; doi:10.3389/fonc.2025.1590877)
Supplement: Supplementary file 1 [file Table1.docx]

**Supplementary Material**

**Supplementary Table 1：Surveillance and follow up of patients with CNC**

Any patient with the diagnosis of CNC should be followed closely for clinical manifestations of the disease at least once a year. A study has shown that this type of follow up improves prognosis.

The suggestion include:

1. Annual echocardiogram, beginning in infancy; if a patient was diagnosed with a cardiac myxoma at least once, cardiac imaging may be done biannually.
2. Regular skin evaluations
3. Blood tests to check serum levels of GH, prolactin, and IGF-1 beginning in adolescence, as appropriate for the detection of GH and PRL excess; urinary free cortisol (UFC) and other testing for screening of Cushing’s syndrome, as appropriate.
4. Thyroid gland (neck) clinical examinations and with ultrasound, if needed.
5. Imaging may include adrenal computed tomography for the detection of PPNAD; pituitary magnetic resonance imaging (MRI), and MRI of brain, spine, chest, abdomen, retroperitoneum, pelvis for the detection of PMS .
6. In males, testicular examinations with ultrasound may be done annually for the detection and follow up of LCCSCT.
7. In females, transabdominal ultrasound of the ovaries (baseline examination; it may be repeated, as needed).
8. In pre-pubertal children: close monitoring of linear growth rate and annual pubertal staging.

**Supplementary Table 2：Diagnostic criteria for CNC**

| ***Major Criteria*** |
| --- |
| 1. Spotty skin pigmentation with typical distribution (lips, conjunctiva and inner or outer canthi, vaginal and penile mucosal) 2. Myxoma[**](https://pmc.ncbi.nlm.nih.gov/articles/PMC4553126/#TFN2) (cutaneous and mucosal) or cardiac myxoma[**](https://pmc.ncbi.nlm.nih.gov/articles/PMC4553126/#TFN2) 3. Breast myxomatosis[**](https://pmc.ncbi.nlm.nih.gov/articles/PMC4553126/#TFN2) or fat-suppressed magnetic resonance imaging findings suggestive of this diagnosis 4. PPNAD[**](https://pmc.ncbi.nlm.nih.gov/articles/PMC4553126/#TFN2) or paradoxical positive response of urinary glucocorticosteroid excretion to dexamethasone administration during Liddle’s test 5. Acromegaly as a result of growth hormone (GH)-producing adenoma[*](https://pmc.ncbi.nlm.nih.gov/articles/PMC4553126/#TFN1) 6. LCCSCT[**](https://pmc.ncbi.nlm.nih.gov/articles/PMC4553126/#TFN2) or characteristic calcification on testicular ultrasound 7. Thyroid carcinoma[*](https://pmc.ncbi.nlm.nih.gov/articles/PMC4553126/#TFN1)(at any age) or multiple hypoechoic nodules on thyroid ultrasound in prepubertal child 8. Psammomatous melanotic schwannomas (PMS)[**](https://pmc.ncbi.nlm.nih.gov/articles/PMC4553126/#TFN2) 9. Blue nevus, epithelioid blue nevus (multiple)[**](https://pmc.ncbi.nlm.nih.gov/articles/PMC4553126/#TFN2) 10. Breast ductal adenoma (multiple)[**](https://pmc.ncbi.nlm.nih.gov/articles/PMC4553126/#TFN2) 11. Osteochondromyxoma[**](https://pmc.ncbi.nlm.nih.gov/articles/PMC4553126/#TFN2) |
| ***Supplemental criteria*** |
| 1. Affected first-degree relative 2. Activating pathogenic variants of PRKACA (single base substitutions and copy number variation) and PRKACB (Beuschlein, Fassnacht et al. 2014, Forlino, Vetro et al. 2014) 3. Inactivating mutation of the PRKAR1A gene (Bossis, Voutetakis et al. 2004) |

^*^To make the diagnosis of CNC, a patient must either: **(1) exhibit two of the major criteria confirmed by histology, imaging or biochemical testing or meet (2) one major criterion and one supplemental one**

^**^with histologic confirmation
